# DPD Study on the Interfacial Properties of PEO/PEO-PPO-PEO/PPO Ternary Blends: Effects of Pluronic Structure and Concentration

**DOI:** 10.3390/polym13172866

**Published:** 2021-08-26

**Authors:** Dongmei Liu, Meiyuan Yang, Danping Wang, Xueying Jing, Ye Lin, Lei Feng, Xiaozheng Duan

**Affiliations:** 1 School of Science, North China University of Science and Technology, Tangshan 063210, China; dmliu@ncst.edu.cn (D.L.); yangmeiyuan777@163.com (M.Y.); danping@ncst.edu.cn (D.W.); jingxueying002@163.com (X.J.); feng_lei2000@ncst.edu.cn (L.F.); 2 State Key Laboratory of Polymer Physics and Chemistry, Changchun Institute of Applied Chemistry, Chinese Academy of Sciences, Changchun 130022, China

**Keywords:** dissipative particle dynamics, interfacial property, Pluronics

## Abstract

Using the method of dissipative particle dynamics (DPD) simulations, we investigated the interfacial properties of PEO/PEO-PPO-PEO/PPO ternary blends composed of the Pluronics L64(EO_13_PO_30_EO_13_), F68(EO_76_PO_29_EO_76_), F88(EO_104_PO_39_EO_104_), or F127(EO_106_PO_70_EO_106_) triblock copolymers. Our simulations show that: (i) The interfacial tensions (γ) of the ternary blends obey the relationship $\gamma_{F68}$ < $\gamma_{L64}$ < $\gamma_{F88}$ < $\gamma_{F127}$, which indicates that triblock copolymer F68 is most effective in reducing the interfacial tension, compared to L64, F88, and F127; (ii) For the blends of PEO/L64/PPO and the F64 copolymer concentration ranging from *c*_cp_ = 0.2 to 0.4, the interface exhibits a saturation state, which results in the aggregation and micelle formation of F64 copolymers added to the blends, and a lowered efficiency of the L64 copolymers as a compatibilizer, thus, the interfacial tension decreases slightly; (iii) For the blends of PEO/F68/PPO, elevating the Pluronic copolymer concentration can promote Pluronic copolymer enrichment at the interfaces without forming the micelles, which reduces the interfacial tension significantly. The interfacial properties of the blends contained the PEO-PPO-PEO triblock copolymer compatibilizers are, thus, controlled by the triblock copolymer structure and the concentration. This work provides important insights into the use of the PEO-PPO-PEO triblock copolymer as compatibilizers in the PEO and PPO homopolymer blend systems.

## 1. Introduction

Pluronics are a non-ionic poly(ethylene oxide)-poly(propylene oxide)-poly(ethylene oxide) (PEO_n_-PPO_m_-PEO_n_, where n and m are the numbers of the repeating units PEO and PPO, respectively) triblock copolymers, also known as poloxamers [1]. PEO-PPO-PEO triblock copolymers that exhibit the dual properties of polymers and surfactants have been widely applied in many fields, such as cosmetics, emulsification, foaming, and the pharmaceutical industry [2,3,4,5,6]. Recent studies on PEO-PPO-PEO triblock copolymers involve the application of Pluronics in the development and application of more complex systems. For example, the application of PEO-PPO-PEO triblock copolymers in gene therapy is one of the promising research topics [7]. Additionally, PEO-PPO-PEO triblock copolymers also exhibit better biocompatibility, which promotes their application in the development of food [8,9,10] and drugs with new and improved long-circulating properties [11]. Since most of their applications are related to their interfacial properties and micelle formation, to rationally design biocompatible composite materials, it is important to better understand the interfacial properties of the blend composed by PEO-PPO-PEO triblock copolymers.

In recent decades, interfacial behaviors of the Pluronic copolymers have received increased attention [12,13,14,15,16,17,18]. In experiments, Welge and Wolf studied the dependence of the reduction of interfacial tension between the homopolymer PEO and PPO on the addition of the Pluronic copolymers by the pendant drop method [12]. They found that the reduced interfacial tension depends, mainly, on the phase that the compatibilizer was added. As the compatibilizers were added to the PPO phase, the efficiency of the triblock copolymers was approximately twice of adding to the PEO phase. Pérez et al. systematically characterized the interfacial behaviors of the PEO21-PPO50-PEO21 (PE9400) at the limonene-water blends [1] and illustrated that the PE9400 triblock copolymers are irreversibly adsorbed on the interface of the blends regardless of the properties of the interface and the configuration of the triblock copolymers. Vidhi Shah et al. investigated the diffusion and adsorption behaviors of the Pluronic copolymers at oil–water interfaces. Their experiments showed that the interfacial tension significantly reduced with the addition of the Pluronic copolymers [13]. They also found that the interfacial tensions obey the following relationship $\gamma_{L62}$ < $\gamma_{L64}$ < $\gamma_{F68}$ < $\gamma_{F87}<\gamma_{F88}$.

Due to the limitations of experimental conditions and capital costs, simulation studies have been extensively performed to probe the morphology and mechanical properties of blends containing the PEO-PPO-PEO triblock copolymers at the molecular level. Sun and co-workers [14] employed DPD simulation to explore the interfacial properties of oil/PEO-PPO-PEO/water blends. They found that the higher the PO/EO ratio and the larger molecular weight of the Pluronic copolymers the lower the interfacial tension, and as the PO/EO ratio is fixed, the interfacial tension and interfacial width increase with the increasing PEO-PPO-PEO molecular weight. Song et al. studied the effect of the triblock copolymer architecture on the adsorption of the Pluronic copolymers at a surface that is hydrophobic by DPD simulation [15] and found that the ability of self-assembly of the Pluronic copolymers on the surface increases with the value of the EO ratio of the triblock copolymer.

Although these studies have deepened our understanding of the interfacial behaviors of Pluronic copolymers, the dependence of the morphology and mechanical properties of the blends on the microscopic configuration of the Pluronic copolymers remains unclear. In this work, we explored the interfacial properties of PEO/PEO-PPO-PEO/PPO ternary blends composed by the L64(EO_76_PO_29_EO_76_), F68(EO_13_PO_30_EO_13_), F88(EO_104_PO_39_EO_104_), or F127(EO_106_PO_70_EO_106_) triblock copolymers using DPD simulations. We first introduce the model and simulation details used in this work. Then, we analyze the interfacial and structural properties by discussing the dependence of the interfacial tension, the density distribution of PEO, PPO homopolymers, and PEO-PPO-PEO copolymers, and the detailed molecular conformations of the Pluronic copolymers on the structure and concentration of the Pluronic copolymers. Our work elucidates that the obtained interfacial tensions obey the following relationship $\gamma_{F68}$ < $\gamma_{L64}$ < $\gamma_{F88}$ < $\gamma_{F127}$. Further, we clarify the effects of the L64 and F68 copolymer concentration on the interfacial and structural properties of the ternary blends. Finally, we briefly summarize our results and offer some concluding remarks.

## 2. Method

We constructed the model of this work based on our previous studies and other studies, refs. [19,20,21,22,23,24,25,26,27,28,29,30,31,32,33,34,35,36]. Interested readers could refer to the details in these works. Herein, we briefly introduce this model.

### 2.1. Model

The DPD method, firstly introduced by Hoogerbrugge and Koelman in 1992 [37,38], is a powerful simulation technology on longer length and time scales compared to the traditional molecular dynamics approach. In a DPD simulation system, a coarse-grained bead, generally, consists of several atoms or molecules interacting through a soft potential. The motion of each bead is governed by Newton’s second law:

(1) $$\frac{dr_{i}}{dt}=v_{i};\;m_{i}\frac{dv_{i}}{dt}=f_{i}$$

where $r_{i}$, $v_{i}$ and $m_{i}$ are the position vector, velocity vector, and mass of the *i*th bead, respectively. The mass $m_{i}$ is normalized to 1. The effective force $f_{i}$ acting on the *i*th bead consists of the conservative force $F_{ij}^{C}$, dissipative force $F_{ij}^{D}$, random force $F_{ij}^{R}$, and harmonic spring force $F_{i}^{S}$, which can be written as follows:

(2) $$f_{i}=\underset{j\neq i}{\sum }\left(F_{ij}^{C}+F_{ij}^{D}+F_{ij}^{R}\right)+F_{i}^{S}$$

The conservative force is generally expressed as:

(3) $$F_{ij}^{C}=-\alpha_{\mathrm{EP}}\omega^{C}\left(r_{ij}\right)e_{ij}$$

where $\alpha_{\mathrm{EP}}$ is the repulsion force parameter, which is the most crucial parameter that determines the force field for beads participating in the simulation. The distance between the *i*th bead and the *j*th bead is $r_{ij}$, which is equal to the absolute value of the vector $r_{ij}=r_{i}-r_{j}$, i.e., $r_{ij}=\left|r_{ij}\right|$. $e_{ij}=r_{ij}/r_{ij}$ is the unit vector. In DPD simulation, we take the conservation force function $\omega^{C}\left(r_{ij}\right)=1-r_{ij}\;\mathrm{for}\;r_{ij}<1\;\mathrm{and}\;\omega^{C}\left(r_{ij}\right)=0\;\mathrm{for}\;r_{ij}\geq 1$ [39].

The dissipative force $F_{ij}^{D}$ and the random force $F_{ij}^{R}$ usually follow:

(4) $$F_{ij}^{D}=-\gamma \omega^{D}\left(r_{ij}\right)\left(v_{ij}\cdot e_{ij}\right)e_{ij}$$

(5) $$F_{ij}^{R}=\sigma \omega^{R}\left(r_{ij}\right)\xi_{ij}\Delta t^{-1/2}e_{ij}$$

where $v_{ij}=v_{i}-v_{j}$, γ is the dissipation (friction coefficient) between beads, σ and $\xi_{ij}$ are the amplitude of the noise and Gaussian random number with zero mean and unit variance. $\omega^{D}\;\mathrm{and}$ $\omega^{R}$ are weight functions for the dissipative forces and the random forces, respectively. The weight functions $\omega^{D}\left(r_{ij}\right)$ and $\omega^{R}\left(r_{ij}\right)$ are chosen of following the fluctuation-dissipation theorem [39]:

(6) $$\omega^{D}\left(r\right)={\left[\omega^{R}\left(r\right)\right]}^{2},\;\sigma^{2}=2\gamma k_{B}T$$

where $k_{B}$ and *T* are the Boltzmann constant and the temperature. All simulations were performed in NVT ensemble with $k_{B}T=1$. According to the research of Groot and Warren [39], the weight functions $\omega^{D}\left(r_{ij}\right)$ and $\omega^{R}\left(r_{ij}\right)$ can be simply chosen of the following form:

(7) $$\omega^{D}\left(r\right)={\left[\omega^{R}\left(r\right)\right]}^{2}=\{\begin{array}{c} \begin{array}{cc} {\left(1-r\right)}^{2} & \left(r<1\right) \end{array}\\ \begin{array}{cc} 0 & \left(r\geq 1\right) \end{array} \end{array}$$

The conservative repulsion interaction parameter $\alpha_{\mathrm{EP}}$ is determined by the following equation [39]:

(8) $$\alpha_{\mathrm{EP}}\approx \alpha_{\mathrm{EE}}+3.50\chi_{\mathrm{EP}}$$

where $\chi_{\mathrm{EP}}$ is the Flory–Huggins parameter between different beads. The repulsion parameters between similar beads are set as $\alpha_{\mathrm{EE}}=\alpha_{\mathrm{PP}}=25$.

The harmonic spring force is introduced to link the adjacent beads on a polymer backbone together:

(9) $$F_{i}^{S}=\sum_{j\neq i}Cr_{ij}$$

where *C* = 4.0 is the corresponding spring force constant.

### 2.2. Simulation Details

In this work, all the DPD simulations were accomplished in a 30 × 30 × 30 cubic box with periodic boundary conditions using Materials Studio (Accelrys Inc., San Diego, CA, USA) software. The cutoff distance of the interaction is $r_{c}=1$. The number density of the system is taken as ρ=3. The repulsion parameter of different types of beads is set as 48.9 [40]. The time step and the friction coefficient γ are taken as 0.05 and 4.5, respectively. The number of beads in a DPD molecule could be calculated by the degree of polymerization and the characteristic ratio of the real polymers, and obey the following relationship [41,42]:

(10) $$N_{\mathrm{DPD}}=\frac{M_{p}}{M_{m}C_{n}}$$

where $M_{p}$ and $M_{m}$ represent the molecular weight of polymer and monomer, respectively. $C_{n}$ is the characteristic ratio, which can be obtained according to quantitative structure–property relationship methods [41]. For the PEO and PPO homopolymer, the estimated $C_{n}$ are 4.98 and 3.26, respectively. The structures [12,13,14] and the corresponding DPD chains of the species in the PEO/PEO-PPO-PEO/PPO blends are listed in Table 1.

In this PEO/PEO-PPO-PEO/PPO ternary blend system, to study the effects of the triblock copolymer structure on the interfacial and structural properties, we calculated the interfacial tension, the density distribution of PEO, PPO, homopolymers, and PEO-PPO-PEO copolymers, and the conformation of the PEO-PPO-PEO copolymers for the ternary blends of E_46_/L64(EO_13_PO_30_EO_13_)/P_13_, E_46_/F68(EO_76_PO_29_EO_76_)/P_13_, E_46_/F88(EO_104_PO_39_EO_104_)/P_13_, E_46_/F127(EO_106_PO_70_E_106_)/P_13_ at the triblock copolymer concentration *c*_cp_ = 0.4. To investigate the effects of PEO-PPO-PEO triblock copolymer concentration on the structural and interfacial properties, we changed the concentration from *c*_cp_ = 0.1 to 0.4 of the triblock copolymer for the blends of E_46_/L64/P_13_ and E_46_/F68/P_13_.

As illustrated by the previous studies, the exact equilibrium location of phase boundaries in the phase separation can be precisely determined by adding a bias to the potential energy of the system to sample different configurations or choosing larger repulsion parameters for the different types of beads to lower the free energy barrier. [43,44] In this work, we mainly focus on the interfacial thermodynamic properties, and it should be noted that the phase transition points (such as the temperature, pressure, and copolymer concentration) are not the exact equilibrium transition conditionist. To save the computational cost and speed up the formation of the interfaces, the PEO, PPO homopolymers, and PEO-PPO-PEO triblock copolymers were initially placed in distinct locations along the x-direction in the box, which can greatly enhance computing efficiency. [20] The simulations were first carried out for 2.0 × 10^5^ steps, which have been proven long enough for the system equilibration [24,25,45]. In each simulation, we calculated the interfacial and structural properties of polymers from 50 structures (with each one selected at the end of every 1000 steps) in the final 5 × 10^4^ steps of the equilibrated state. To obtain more accurate results, for each case, we carried out several parallel simulations and provided the ultimate average results in the paper. The comprehensive sample-to-sample fluctuations in each simulation and from different simulation trajectories are shown by the error bars.

In a ternary blend system, the interfacial tension is one of the key factors to evaluate the interfacial and structural properties of the blends. In DPD simulation, according to the Virial theorem [46], if the stresses normal to the interfaces are parallel to the x-axis, the interfacial tension can be calculated by the following formula [30]:

(11) $$\gamma_{\mathrm{DPD}}=\frac{1}{2}L\left[\langle P_{\mathrm{xx}}\rangle -\frac{1}{2}\left(\langle P_{\mathrm{yy}}\rangle +\langle P_{\mathrm{zz}}\rangle \right)\right]$$

where *P*_xx_ is the pressure tensor along the x-axis (normal to the interface), and *P*_yy_ and *P*_zz_ are the pressure tensor along the y-axis and z-axis (parallel to the interface), respectively. < > is the ensemble average of the pressure tensor components and *L* is the length of the simulation box in the *x*-direction. In addition, to characterize the detailed Pluronic copolymer conformations, we calculated the mean-square radius of gyration <*R*_g_^2^> and its three components <*R*_g(x)_^2^>, <*R*_g(y)_^2^>, <*R*_g(z)_^2^>, the mean-square end-to-end distance <*R*_ee_^2^> and its three components <*R*_ee(x)_^2^>, <*R*_ee(y)_^2^>, <*R*_ee(z)_^2^>, and the orientation parameter referring to the study of Qian et al. [20]. The orientation parameter is determined as follows:

(12) $$q=\;\frac{\left(\langle R_{g\left(x\right)}^{2}\rangle -1/2\left(\langle R_{g\left(y\right)}^{2}\rangle +\langle R_{g\left(z\right)}^{2}\rangle \right)\right)}{\langle R_{g}^{2}\rangle }$$

where *q* is the chain orientation parameter, which denotes the size difference between x and y, z components of the triblock copolymers.

## 3. Results and Discussion

### 3.1. Effect of the Pluronic Copolymer Structure

**Figure 1:**
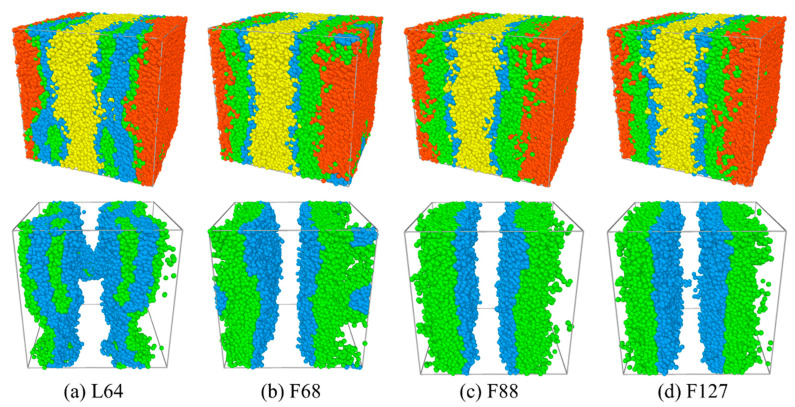
Morphology snapshots for ternary blends of (**a**) E_46_/L64/P_13_, (**b**) E_46_/F68/P_13_, (**c**) E_46_/F88/P_13_, and (**d**) E_46_/F128/P_13_ at copolymer concentration of *c*_cp_ = 0.4. The red and yellow spheres denote bead E and bead P of homopolymers E_46_ and P_13_, and the green and blue spheres represent beads E and P of the Pluronic.

**Figure 2:**
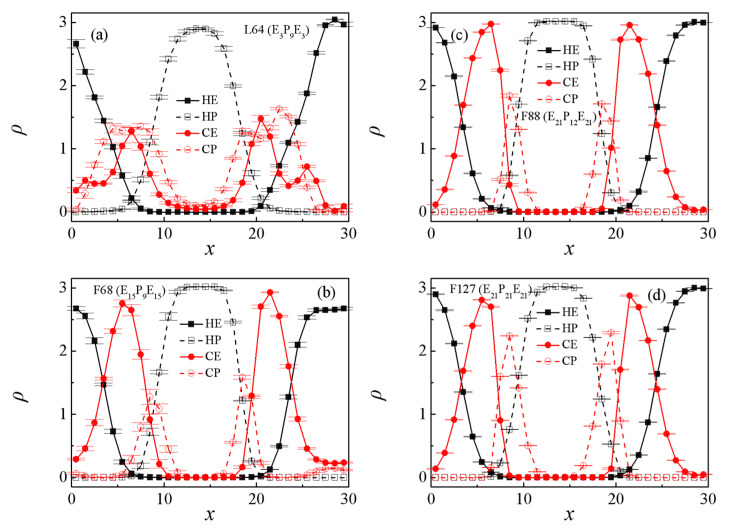
Density profiles of beads E and P of the PEO, PPO homopolymer, and PEO-PPO-PEO triblock copolymers along the *x*-axis for the blends of (**a**) E_46_/L64/P_13_, (**b**) E_46_/F68/P_13_, (**c**) E_46_/F88/P_13_, and (**d**) E_46_/F128/P_13_ at copolymer concentration of *c*_cp_ = 0.4.

In this section, the effects of the structure of the Pluronic copolymer on the interfacial properties are analyzed and discussed. Figure 1 and Figure 2 show the morphology snapshots, the density profiles of beads E, P of PEO, PPO homopolymers, and PEO-PPO-PEO copolymers for the blends of E_46_/L64/P_13_, E_46_/F68/P_13_, E_46_/F88/P_13_, and E_46_/F127/P_13_, respectively. The concentration of the Pluronic copolymers is set as *c*_cp_ = 0.4. We found that most Pluronic copolymers are segregated into the PEO/PPO homopolymer interface, which leads to a decayed correlation between the PEO and PPO homopolymers. It can be seen that most L64 and F68 copolymers segregate into the PEO/PPO interface, and the rest small part of L64 and F68 copolymers aggregate at the PEO or PPO homopolymer phase, which indicates that the interfaces of the blends E_46_/L64/P_13_ and E_46_/F68/P_13_ have reached saturation (as illustrated in Figure 1a,b). Figure 1a also shows that the L64 copolymers form micelles with the core composed of E beads of the L64 copolymers (the structure of the L64 copolymers is depicted in Figure S1a in the Electronic Supplementary Information). The density profile of beads E of the L64 copolymers exhibits two peaks between the PEO and PPO homopolymer phase (the red solid dots at 0 < *x* < 10 and 20 < *x* < 30 of Figure 2a), which corresponds to the formation of unsmooth interfaces shown by the snapshots in Figure 1a. Specifically, the higher peaks of the density profile of beads E around *x*~7.5 or *x*~21.5 closed to the PPO homopolymer phase indicate the core of micelles formed by L64 copolymers at the interfaces. Specifically, the L64 copolymers form a “hairpin loop”-type of conformation near the PPO homopolymer phase, the rest of the L64 copolymers form a “bridge”-type of conformation, which is in good agreement with the experimental observations of Kramer et al. [47].

However, in the blends of E_46_/F68/P_13_, E_46_/F88/P_13_, and E_46_/F127/P_13_, the central P beads of the F68, F88, and F127 copolymers preferentially segregate into the PPO homopolymers bulk phase, and both the end E beads segregate into the PEO homopolymer bulk phase (see Figure 1b–d and Figure 2b–d), which indicates that the F68, F88, and F127 copolymers form a “hairpin loop”-type of conformation at the interface (as illustrated in Figure S1b). It is noted that the micelles in Figure 1a,b exhibit different structures. As demonstrated by Kramer et al. [47], the micelles that the core composed of smaller block exhibits lower copolymer chemical potential. Likewise, in our simulation, the two end E blocks of L64 copolymers in Figure 1a are shorter than the center P blocks, and the micelles formed in the PPO phase at the lowered chemical potential. On the contrary, the end E blocks of F68 copolymer in Figure 1b are larger than the center P block, thus, the cores of the formation micelles in the PEO phase are composed of the center P block, which results in a lower segregation rate for F68 than the F88 and F127 copolymers [Figure 1c,d].

**Figure 3:**
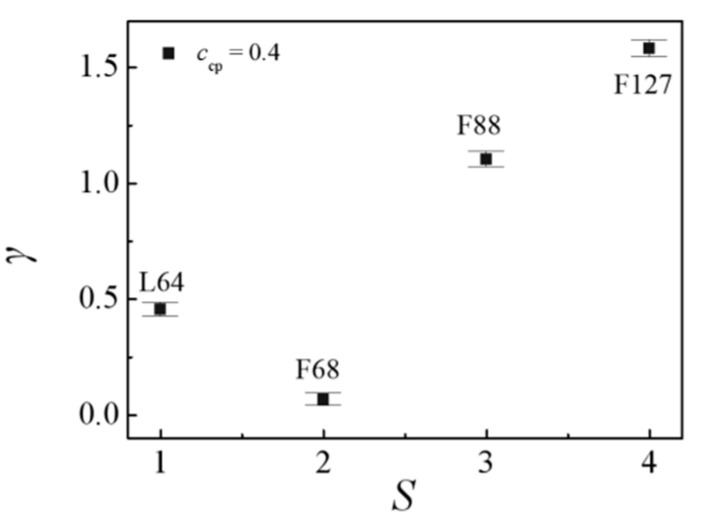
Interfacial tension γ as a function of Pluronic copolymer structure at copolymer concentration of *c*_cp_ = 0.4 (the Pluronic copolymer structure *S* = L64, F68, F88, F127).

Figure 1 shows that the segregation of the Pluronic copolymers at the PEO/PPO homopolymer interface strongly depends upon the triblock copolymer structure. To quantitatively study the effects of the structure of the Pluronic copolymers on the interfacial properties, we calculated the interfacial tension γ for the blends of Pluronic copolymers with different structures, as shown in Figure 3. Herein, we found that the interfacial tensions γ of the blends obey the following relationship E_46_/F68/P_13_ < E_46_/L64/P_13_ < E_46_/F88/P_13_ < E_46_/F127/P_13_. However, the experimental research by Zhao indicated that the shorter the triblock copolymer chain length added to the immiscible homopolymers blend the smaller the interfacial tension γ [48]. We inferred that the larger interfacial tension γ for the blends of E_46_/L64/P_13_ can be attributed to the formation of the micelles (as illustrated in Figure 1a and Figure 2a). As illustrated by the previous studies, the formation of micelles can cause the decrease in the chemical potential of copolymers that drive the segregation, leading to the decrease in the kinetics of segregation [49] and result in the reduced rate of the ordering process [50]. Hence, the micellization lowers the efficiency of the L64 triblock copolymers as a compatibilizer. Ref. [47]. As the structure of Pluronic copolymer varies from F68 to F127, the chain length of the Pluronic copolymers increases, which leads to the number of the Pluronic copolymers at the interface per area decreasing [20], thus, the interfacial tension γ increases. These findings indicate that the efficiency of the Pluronic copolymers in maintaining the stability of the blends is ranked as F68 > L64 > F88 > F127.

**Figure 4:**
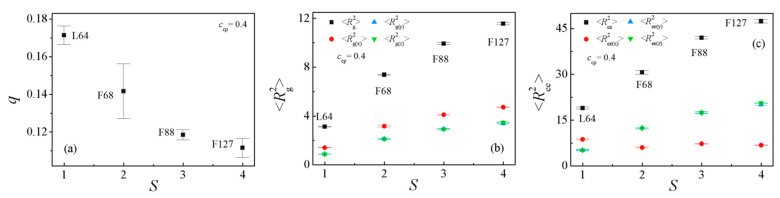
(**a**) Orientation parameter of Pluronic copolymers, (**b**) mean squared radius gyration <*R*_g_^2^> and the three components <*R*_g(x)_^2^>, <*R*_g(y)_^2^>, <*R*_g(z)_^2^>, and (**c**) mean-squared end-to-end distance <*R*_ee_^2^> and the three components <*R*_ee(x)_^2^>, <*R*_ee(y)_^2^>, <*R*_ee(z)_^2^> as a function of the Pluronic copolymer structure with *c*_cp_ = 0.4 (the Pluronic copolymer structure *S* = L64, F68, F88, F127).

To examine the effects of the structure of Pluronic copolymers on the detailed molecular conformation of the Pluronic copolymers at the interface, we calculated the chain orientation parameter *q* and dimension (the mean-square radius gyration <*R*_g_^2^> and its three components <*R*_g(x)_^2^>, <*R*_g(y)_^2^>, <*R*_g(z)_^2^>, the mean-square end-to-end distance <*R*_ee_^2^> and its three components <*R*_ee(x)_^2^>, <*R*_ee(y)_^2^>, <*R*_ee(z)_^2^>) of the Pluronic copolymers. Figure 4 shows the dependence of the chain orientation parameter *q* and dimension of the Pluronic copolymers on the structure. The orientation parameter *q* decreases when the structure of the Pluronic copolymers varies from L64 to F127 [Figure 4a], implying that the shorter L64 copolymers are more stretched in the direction perpendicular to the interface. Figure 4b shows that the mean-square radius gyration <*R*_g_^2^> and its three components <*R*_g(x)_^2^>, <*R*_g(y)_^2^>, <*R*_g(z)_^2^> increase when the structure of the Pluronic copolymers varies from L64 to F127, which are mainly related to the increase of the Pluronic copolymer chain length. In addition, it is found that the <*R*_ee(y)_^2^> and <*R*_ee(z)_^2^> of the L64 copolymers along the *y* and *z* directions are smaller than the <*R*_ee(x)_^2^> along the x-direction, and the <*R*_ee(x)_^2^> of the L64 copolymers is larger than the <*R*_ee(x)_^2^> of the F68, F88, F127 copolymers [Figure 4c]. These results are due to the L64 copolymers at the interface forming micelles, and the L64 copolymers of the micellization exhibiting the “bridge”-type of conformation, whereas the F68, F88, and F127 copolymers at the interface form the folded “hairpin loop”-type of conformation, thus, the <*R*_ee(x)_^2^> is larger than <*R*_ee(y)_^2^> and <*R*_ee(z)_^2^> of L64, and the orientation parameter *q* and <*R*_ee(x)_^2^> of the L64 copolymers are larger than that of the F68, F88, and F127 copolymers.

Since we have demonstrated that the L64 and F68 triblock copolymers exhibit better performance in reducing the interfacial tension γ at the interface of the PEO/PEO-PPO-PEO/PPO ternary blends, we further focus on exploring the effects of the L64 and F68 copolymers concentration on the interfacial properties and the detailed conformation of the Pluronic copolymers.

### 3.2. Effect of Pluronic Copolymer Concentration

**Figure 5:**
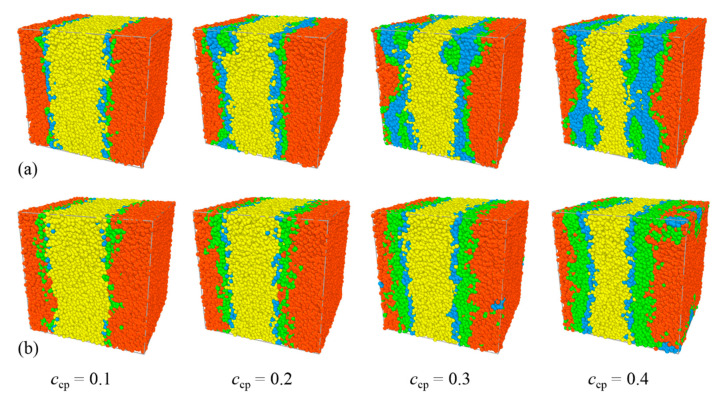
Morphology snapshots for ternary blends at different PEO-PPO-PEO concentrations. Compositions are (**a**) E_46_/L64/P_13_ and (**b**) E_46_/F68/P_13_. The red and yellow spheres denote bead E and bead P of homopolymers E_46_ and P_13_, and the green and blue spheres represent beads E and P of the Pluronic.

**Figure 6:**
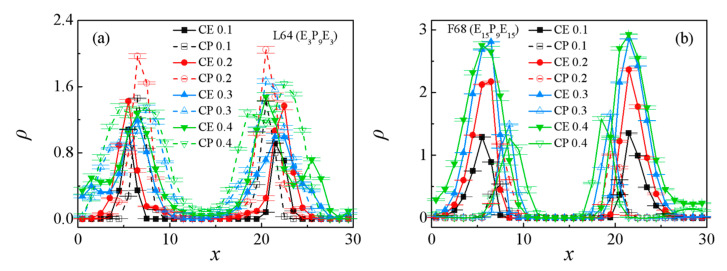
Density profiles of beads E and P of the Pluronics along the *x*-axis as a function of Pluronic copolymer concentration with (**a**) L64 and (**b**) F68.

We further explore the dependence of the interfacial and structural properties on the Pluronic copolymers concentration *c*_cp_. Figure 5 and Figure 6 show the morphology snapshots and density of beads E and P of Pluronic copolymer for the blends of E_46_/L64/P_13_ and E_46_/F68/P_13_, respectively. In the case of E_46_/L64/P_13_ blends, as the concentration *c*_cp_ of L64 copolymer increases from 0.1 to 0.2, the density of beads E and P of L64 copolymers at the interface increases (see Figure 5a, the black squares and red dots in Figure 6a), and the L64 triblock copolymers begin to form micelles at the interface; as *c*_cp_ of L64 copolymer increases from 0.2 to 0.3, the density of beads E and P of L64 copolymers near the center of the interface decreases (the blue upper triangle in Figure 6a), the L64 copolymers of the micellization increase; as *c*_cp_ of L64 copolymer further increases to 0.4, the density of beads E and P of L64 copolymers near the center of the interface change slightly, the L64 copolymers of formation micelles further increase. Figure 5 also shows that the formation of micelles mainly depends on the L64 concentration. Specifically, at a low L64 concentration of *c*_cp_ = 0.1, no micelles exist. As the L64 concentration *c*_cp_ increases from 0.2 to 0.4, the small micelles grow to larger clusters at the interface. When the L64 copolymer concentration *c*_cp_ = 0.2, the interface has reached saturation and formed micelles, and as *c*_cp_ of the L64 copolymer further increases to 0.4, the L64 copolymers added to the blend further aggregate into the micelles, thus, the micelles grow from small micelles to larger clusters. However, for the E_46_/F68/P_13_ blends, as the concentration *c*_cp_ of F68 copolymer increases from 0.1 to 0.3, the density of beads E and P of the F68 copolymers at the interface increases (see Figure 5b, the black squares, red dots, and blue upper triangles in Figure 6b); as *c*_cp_ of F68 copolymer further increases to 0.4, the density of beads E and P of the F68 copolymers near the center of the interface remain almost unchanged, whereas the density of beads E and P of the F68 copolymers at the PEO homopolymer bulk phase increases (Figure 5b and the green inverse triangle in Figure 6b).

**Figure 7:**
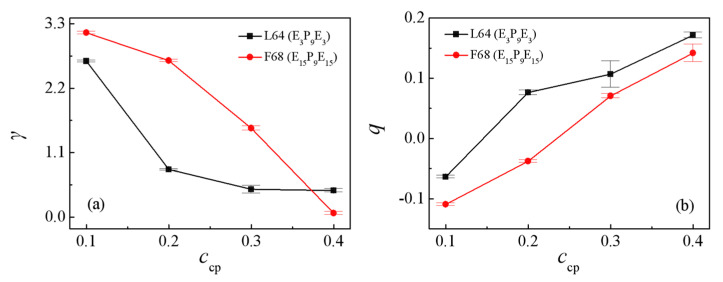
Interfacial tension γ (**a**) and the orientation parameter *q* of the L64 and F68 copolymer (**b**) as a function of L64 and F68 copolymer concentration.

Figure 7a shows the dependence of the interfacial tension γ on the Pluronic copolymers concentration *c*_cp_. For the blends of E_46_/L64/P_13_, the obtained interfacial tension γ rapidly decrease with increasing the L64 copolymer concentration *c*_cp_ from 0.1 to 0.2, this result is because as *c*_cp_ increases from 0.1 to 0.2, the density of beads E and P of the L64 copolymers at the interface increases (as illustrated in Figure 5a and Figure 6a), which results in the decayed correlations between the PEO and PPO homopolymers, thus, the interfacial tension γ decreases. Further, as *c*_cp_ increases from 0.2 to 0.4, the interfacial tension γ decreases slightly. This is because when the L64 copolymer concentration *c*_cp_ = 0.2, the interface has reached saturation and formed micelles, as *c*_cp_ of the L64 copolymer further increases to 0.4, the L64 copolymers added to the blend further aggregate and form micelles at the interface, thus, the interfacial tension γ decreases slightly with increasing L64 copolymer concentration *c*_cp_. However, for the blends of E_46_/F68/P_13_, the interfacial tension γ decreases as the concentration of the F68 copolymer *c*_cp_ increases from 0.1 to 0.4. This result can be interpreted as follows: as the F68 copolymer concentration *c*_cp_ increases, the density of beads E and P of the F68 copolymers at the interface increases without forming the micelles, which results in the decayed correlations between PEO and PPO homopolymer, thus, the interfacial tension γ monotonically decreases.

**Figure 8:**
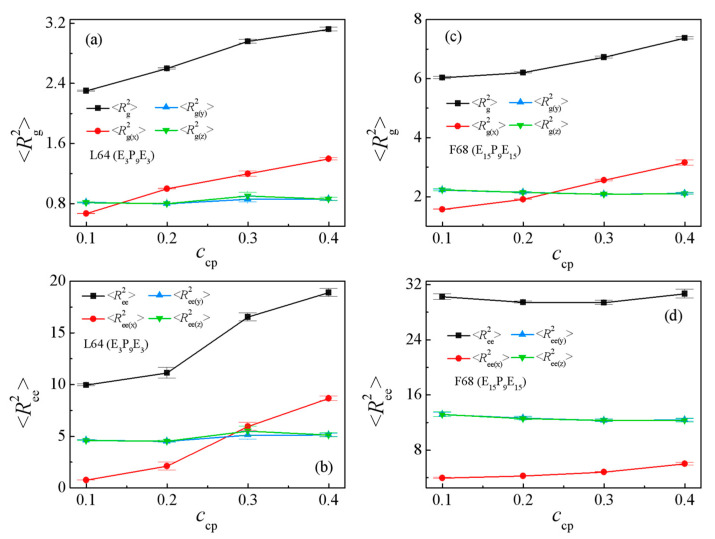
Mean squared radius gyration <*R*_g_^2^> and the three components <*R*_g(x)_^2^>, <*R*_g(y)_^2^>, <*R*_g(z)_^2^> of the Pluronic copolymers as a function of Pluronic copolymer concentration with (**a**) L64 and (**c**) F68. Mean-squared end-to-end distance <*R*_ee_^2^> and the three components <*R*_ee(x)_^2^>, <*R*_ee(y)_^2^>, <*R*_ee(z)_^2^> of the Pluronic copolymers as a function of Pluronic copolymer concentration with (**b**) L64 and (**d**) F68.

Figure 7b and Figure 8 show the dependence of the chain orientation parameter *q* and dimension of the L64 and F68 on their concentration *c*_cp_. We found that as the L64 and F68 concentration increases from *c*_cp_ = 0.1 to 0.4, the orientation parameters *q* of the L64 and F68 increase [Figure 7b], which indicates that the L64 and F68 are more stretched at higher triblock copolymer concentration *c*_cp_ along the *x*-direction (perpendicular to the interface). Figure 8a,c show that the <*R*_g_^2^>, <*R*_g(x)_^2^> of the L64 and F68 increase with increasing the L64 and F68 concentration, whereas the <*R*_g(y)_^2^> and <*R*_g(z)_^2^> remains almost unchanged, which corresponds to the change of the *q*. Figure 8b,d show that the <*R*_ee_^2^> and <*R*_ee(x)_^2^> increase rapidly, the <*R*_ee(y)_^2^> and <*R*_ee(z)_^2^> remains almost unchanged with the increase of triblock copolymer concentration from *c*_cp_ = 0.1 to 0.4 with the L64 system. For the F68 system, the <*R*_ee_^2^> and <*R*_ee(x)_^2^>, <*R*_ee(y)_^2^>, <*R*_ee(z)_^2^> change slightly with the increase of the F68 concentration. These results can be interpreted as follows: in the blends of E_46_/L64/P_13_, the more L64 in the blends the more L64 copolymers the forming micelles, which results in the more L64 copolymers the exhibited “bridge”-type of conformation with larger the <*R*_ee_^2^> and <*R*_ee(x)_^2^>. However, for the E_46_/F68/P_13_ blends, because the two end E blocks of F68 triblock copolymers are always segregated into the PEO homopolymer phase, the central P blocks of the triblock copolymer are always segregated into the PPO homopolymer phase (Figure 5b); that is, the conformation of the triblock copolymer remains “hairpin”-type of conformation unchanged, thus, the <*R*_ee_^2^>, <*R*_ee(x)_^2^>, <*R*_ee(y)_^2^> and <*R*_ee(z)_^2^> of the F68 change slightly.

## 4. Conclusions

In this paper, the effects of the PEO-PPO-PEO triblock copolymer structure and concentration on the interfacial properties of PEO/PEO-PPO-PEO/PPO ternary blends are investigated by the method of dissipative particle dynamics (DPD) simulations.

By comparing the interfacial tension of the E_46_/L64/P_13_, E_46_/F68/P_13_, E_46_/F88/P_13_, and E_46_/F127/P_13_ blends at triblock copolymer concentration *c*_cp_ = 0.4, we found that the interfacial tensions γ of the blends obey the relationship $\gamma_{F68}$ < $\gamma_{L64}$ < $\gamma_{F88}$ < $\gamma_{F127}$, which indicates that the efficiency of the Pluronic copolymers in maintaining the stability of the blends is ranked as F68 > L64 > F88 > F127. We then explored the effect of Pluronic copolymer concentration on the interfacial properties and the detailed conformation of the triblock copolymers at the interfaces. For the E_46_/L64/P_13_ blends, as the triblock copolymer concentration increases from *c*_cp_ = 0.1 to 0.2, the density of beads E and P of L64 copolymer at the interface significantly increases, which results in the reduction of the interfacial tension. Because the interface has reached saturation and formed micelles at the L64 copolymer concentration *c*_cp_ = 0.2, as *c*_cp_ of the L64 copolymer further increases to 0.4, the L64 copolymers added to the blend further aggregate and form micelles with the “bridge”-type of conformation at the interface. Due to the lowered efficiency of the L64 triblock copolymers as a compatibilizer caused by micellization, the interfacial tension γ decreases slightly with increasing L64 copolymer concentration *c*_cp_. For the E_46_/F68/P_13_ blends, by elevating the F68 copolymer concentration from *c*_cp_ = 0.1 to 0.4, the density of the F68 copolymers between the PEO and PPO homopolymers increases without forming the micelles, thus, the interfacial tension decreases monotonically.

Our studies indicate that the interfacial and phase properties of the PEO/PEO-PPO-PEO/PPO ternary blends are strongly correlated to the structure and concentration of the Pluronic copolymer. In this context, it would be interesting to further explore the influence of other molecular parameters in detail on the interfacial and phase properties of the PEO/PEO-PPO-PEO/PPO ternary blend.

## Figures and Tables

**Table 1:** The coarse-grained chains of the polymers in our DPD.

Name	Polymer Structure	Coarse-Grained DPD Chains
PEO	EO_229_	E_46_
PPO	PO_42_	P_13_
L64	EO_13_PO_30_EO_13_	E_3_P_9_E_3_
F68	EO_76_PO_29_EO_76_	E_15_P_9_E_15_
F88	EO_104_PO_39_EO_104_	E_21_P_12_E_21_
F127	EO_106_PO_70_EO_106_	E_21_P_21_E_21_

## Supplementary Materials

The following are available online at https://www.mdpi.com/article/10.3390/polym13172866/s1.
