# Supplementary material for: DPD Study on the Interfacial Properties of PEO/PEO-PPO-PEO/PPO Ternary Blends: Effects of Pluronic Structure and Concentration
Source: Polymers (Basel). 2021 Aug 26;13(17):2866. doi: 10.3390/polym13172866 (PMC8433662; doi:10.3390/polym13172866)
Supplement: Supplementary file 1 [file polymers-13-02866-s001.zip › polymers-1353365-supplementary.pdf]

polymers

MDPI

Article

DPD Study on the Interfacial Properties of PEO/PEO-PPO-PEO/PPO Ternary Blends: Effects of Pluronic Structure and Concentration

Dongmei Liu [1], Meiyuan Yang [1], Danping Wang [1], Xueying Jing [1], Ye Lin [1,*], Lei Feng [1] and Xiaozheng Duan [2,*]

[1] School of Science, North China University of Science and Technology, Tangshan 063210, China; dmliu@ncst.edu.cn (D.L.); yangmeiyuan777@163.com (M.Y.); danping@ncst.edu.cn (D.W.); jingxueying002@163.com (X.J.); feng_lei2000@ncst.edu.cn (L.F.)

[2] State Key Laboratory of Polymer Physics and Chemistry, Changchun Institute of Applied Chemistry, Chinese Academy of Sciences, Changchun 130022, China

* Correspondence: linye315317@163.com (Y.L.); xzduan@ciac.ac.cn (X.D.); Tel.: +86-315-8805860 (Y.L.); +86-431-85262479 (X.D.)

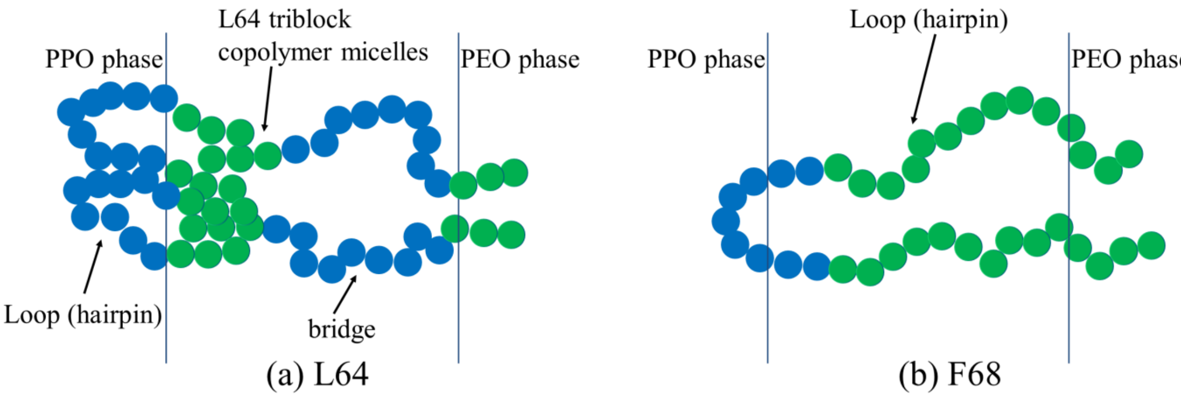

Figure S1. Representative snapshots of the structure for (a) L64 and (b) F68. The green and blue spheres represent beads E and P of the PEO-PPO-PEO copolymers.
